# Retraction: Comprehensive Logic Based Analyses of Toll-Like Receptor 4 Signal Transduction Pathway

**DOI:** 10.1371/journal.pone.0349421

**Published:** 2026-05-18

**Authors:** The PLOS One Editors

The *PLOS One* Editors retract this article [1] due to concerns about compromised peer review.

MKP and US did not agree with the retraction. BS either did not respond directly or could not be reached.
